# Mechanisms of Tolerance Induction by Dendritic Cells *In Vivo*

**DOI:** 10.3389/fimmu.2018.00350

**Published:** 2018-02-26

**Authors:** Hitoshi Hasegawa, Takuya Matsumoto

**Affiliations:** ^1^Department of Hematology, Clinical Immunology and Infectious Diseases, Ehime University Graduate School of Medicine, Toon, Japan

**Keywords:** dendritic cells, immune tolerance, regulatory T cells, development, thymus, skin, intestine

## Abstract

Dendritic cells (DCs) are a heterogeneous population playing a pivotal role in immune responses and tolerance. DCs promote immune tolerance by participating in the negative selection of autoreactive T cells in the thymus. Furthermore, to eliminate autoreactive T cells that have escaped thymic deletion, DCs also induce immune tolerance in the periphery through various mechanisms. Breakdown of these functions leads to autoimmune diseases. Moreover, DCs play a critical role in maintenance of homeostasis in body organs, especially the skin and intestine. In this review, we focus on recent developments in our understanding of the mechanisms of tolerance induction by DCs in the body.

## Introduction

Dendritic cells (DCs) represent a heterogeneous population derived from distinct hematopoietic lineages of bone marrow origin, being characterized by specific homing patterns and specialized immune functions (1–4). DCs play a pivotal role in immune responses and tolerance. Efficient priming of T cells by DCs leading to immune responses requires additional signals from the pro-inflammatory environment that can be sensed by DCs through specific pattern recognition receptors including toll-like receptors (TLRs) (mature DCs; mDCs). In contrast, lack of T cell priming in the absence of pro-inflammatory stimuli initially led to the characterization of DCs as potentially tolerogenic immature bystanders under steady-state conditions (immature DCs; iDCs). Semi-mDCs induced by apoptotic cells, by a special cytokine environment such as IL-10 and TGF-β, or by pharmacological agents also show tolerogenic properties (5–7). Tolerogenic DCs (tolDCs) in the body play an essential role in central and peripheral tolerance, resulting in resolution of ongoing immune responses and prevention of autoimmunity. DCs promote immune tolerance through negative selection of autoreactive T cells and generation of regulatory T cells (Tregs) in the thymus during acquisition of central tolerance. They also limit the differentiation of effector T cells and promote that of Tregs in the periphery through various mechanisms. Breakdown of these functions leads to autoimmune diseases. The skin and intestine act as large barrier organs to the external environment, being exposed to a wide range of environmental antigens such as foods, commensal bacteria, and pathogens. In both organs, DCs fulfill a crucial role in the balance of immune responses, leading to homeostasis and prevention of unnecessary inflammation (8). Accordingly, it is important to analyze the role of DCs in the mechanism of immune tolerance. This review presents an overview of our current understanding of the mechanisms of tolerance induction by DCs in the body.

## DC Origin, Differentiation, and Subsets

**Figure 1:**
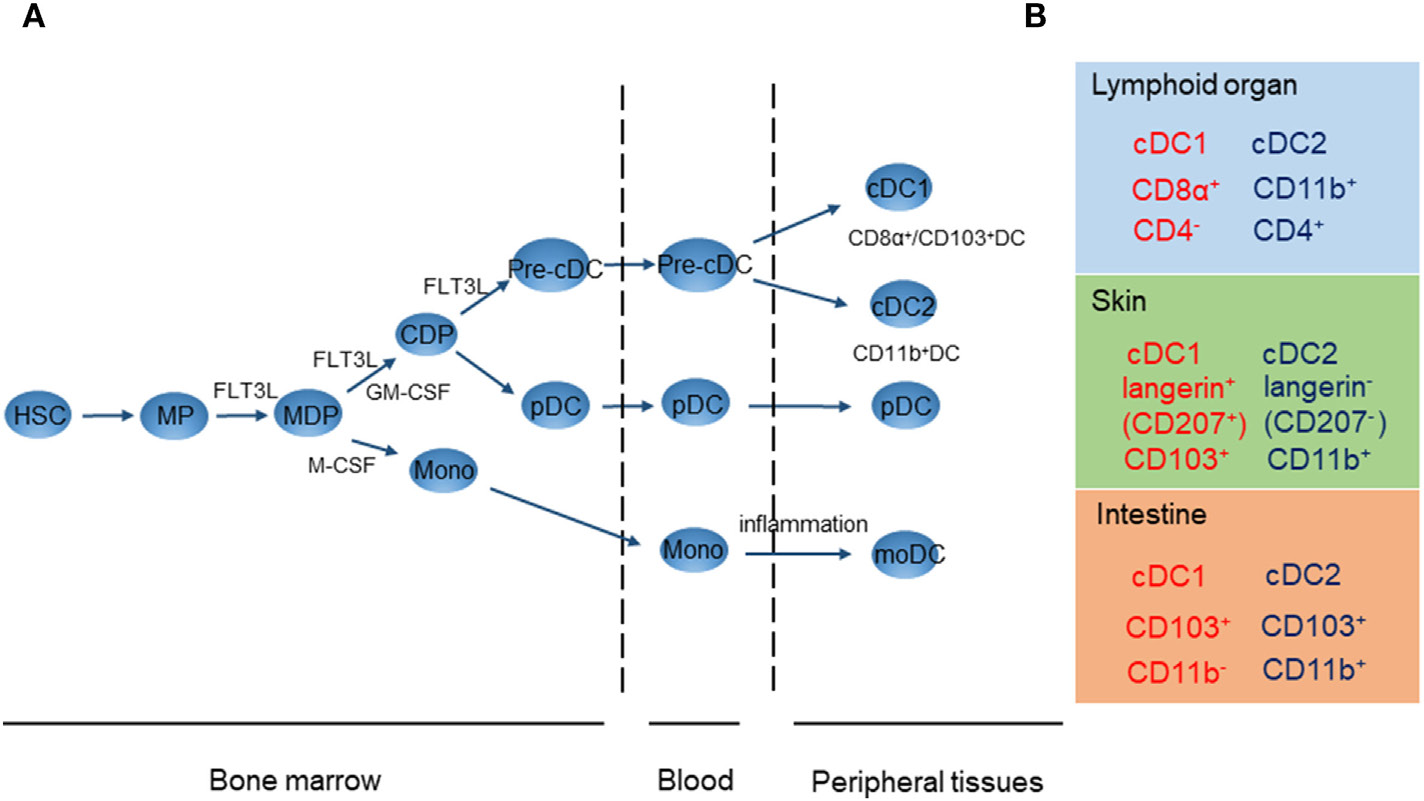
DC development **(A)** and location and phenotypes of mouse conventional DCs 1 (cDC1s) and conventional DCs 2 (cDC2s) **(B)**. **(A)** DC, dendritic cells; HSC, hematopoietic stem cells; MP, myeloid procursor; MDP, macrophage/DC progenitor; CDP, common DC progenitor; cDC, conventional DC; pDC, plasmacytoid DC; moDC, monocyte-derived DC. **(B)** Location and phenotypes of mouse cDC1s (red) and cDC2s (blue).

Dendritic cells originate from CD34^+^ hematopoietic progenitor cells in the bone marrow, which then differentiate further *via* common macrophage/DC progenitors into the monocyte/macrophage lineage or common DC progenitors (CDP) (Figure 1A) (9). CDPs give rise to both plasmacytoid DCs (pDCs) and pre-conventional DC (cDC) progenitors. Fms-like tyrosine kinase 3 ligand (FLT3L) and its receptor, FLT3, have an instructive role in the commitment of hematopoietic progenitors to the DC-restricted lineage and their subsequent development (10, 11). FLT3L is sufficient to drive DC differentiation from mouse and human precursors, since expression of FLT3 is restricted to the DC lineage (11). Before they migrate into the bloodstream, pDCs complete their last step of maturation in the bone marrow before they migrate into the blood stream. Pre-cDC progenitors then migrate through the vascular system to their final locations in tissues or lymphoid organs, before completing their differentiation into iDCs comprising two distinct cDC subsets, CD8α^+^/CD103^+^ DCs [conventional DCs 1 (cDC1s)] and CD11b^+^ DCs [conventional DCs 2 (cDC2s)] (3). On the other hand, monocyte-derived DCs (moDCs) can differentiate from CD14^+^ monocytes under the influence of a combination of stimuli, including GM-CSF, TNF-α, and IL-4, during tissue inflammation (12, 13). DCs are more numerous in lymphoid organs and epithelia and can express various molecular markers depending on their location. Therefore, cDC1s, cDC2s, and pDCs are present in different tissues. Figure 1B shows the cDC cluster to which each cell type belongs. In this context, it is necessary to consider the phenotype and specific location of DCs when addressing their function in particular tissues (9).

In mice, lymphoid organ-resident CD8α^+^ DCs and migratory tissue-resident CD103^+^ DCs have a common origin (9). Their development is dependent on FLT3L, inhibitor of DNA binding protein 2, the transcription factor interferon regulatory factor 8 (IRF8), and the basic leucine zipper transcription factor ATF-like 3 (BATF3) (9). Functional and phenotypic comparison has shown that the human counterpart of murine CD8α^+^/CD103^+^ DCs is CD141 (BDCA-3)-positive DCs (14). CD8α^+^/CD103^+^ DCs share common receptors such as chemokine receptor XCR1 and lectin receptor CLEC9A (15–17). CD8α^+^/CD103^+^ DCs are responsible for efficient cross-presentation of antigen and stimulation of CD8^+^ T cell immunity through secretion of IL-12, thus promoting Th1 differentiation (18, 19). In contrast, in the non-inflamed intestine, CD103^+^ DCs in the lamina propria express high levels of TGF-β and retinaldehyde dehydrogenase 2 (RALDH2), leading to induction of Tregs (20). Therefore, CD8α^+^/CD103^+^ DCs induce either mucosal tolerance or cross-presentation-dependent CD8^+^ T cell immunity on the basis of the local microenvironment.

In mice, CD11b^+^ DCs are present in all major lymphoid and non-lymphoid organs. Development of CD11b^+^ DCs depends on various transcription factors including neurogenic locus notch homolog protein 2, V-Rel avian reticuloendotheliosis viral oncogene homolog B, and IRF4 (9). The human counterpart of murine CD11b^+^ DCs is CD1c (BDCA-1)-positive DCs (21). CD11b^+^ DCs in the spleen express CD4^+^ and can be subdivided according to their expression of the endothelial cell-selective adhesion molecule (22). Splenic CD11b^+^ DCs show higher expression of MHC class II than CD8α^+^ DCs and can present antigen more effectively to CD4^+^ T cells in both the steady state and during inflammation (23). In contrast, CD11b^+^ DCs in the skin and CD11b^+^CD103^+^ DCs in the lamina propria are reported to induce Treg differentiation through retinoic acid (RA) metabolism (20, 24, 25). Both CD8α^+^/CD103^+^ DCs and CD11b^+^ DCs induce tolerance or CD4^+^ T cell proliferation according to the local microenvironment.

Murine pDCs are defined as CD11c^+^, MHC-II^+^, B220^+^/CD45R^+^, BST2^+^, and SiglecH^+^ cells and depend on the transcription factor E2-2 for their development (26). pDCs express high levels of TLR7 and 9, which when ligated by viral products stimulate secretion of a large amount of type I IFN. pDCs can upregulate the expression of MHC class II, allowing the induction of T cell proliferation. On the other hand, murine pDCs induce differentiation of T cells into regulatory type 1 T (Tr1) cells (27). Naïve T cell stimulation using CpG oligonucleotide-stimulated human pDCs has been reported to give rise to Tregs with suppressive properties (28). Phenotypic markers of mouse and human DC subsets are summarized in Table 1.

**Table 1:** Phenotypic markers of mouse and human dendritic cell (DC) subsets.

DC subset	Conventional DCs 1	Conventional DCs 2	Plasmacytoid DC
Mouse	CD8α^+^/CD103^+^ DCs	CD11b^+^ DCs	

Human	CD141^+^ DCs	CD1c^+^ DCs	

Markers			
Common (mouse and human)	BTLA^+^	BTLA^+^	BTLA^+^
	MHCII^+^	MHCII^+^	MHCII^+^
	CD45^+^	CD45^+^	CD45^+^
	CD14^−^	CD14^−^	CD14^−^
	CD11c^+^	CD11c^+^	CD45RA^+^
	CCR7^+^	CCR7^+^	CD123^+^
	FLT3^+^	FLT3^+^	CD4^+^
	CD26^+^	CD4^+^	
	XCR1^+^	CX3CR1^+^	
	CLEC9A^+^	CD11b^+^	
	TLR3^+^	SIRPα^+^	

Mouse only	CD8α^+^	CD24^+^	B220/CD45R^+^
	CD103^+^		BST2^+^
	CD205^+^		Ly6C^+^
	Langerin^+^		SiglecH^+^

Human only	CD4^+^	CD1a^+^	CD303^+^
	CD141^+^	CD1b^+^	CD304^+^
		CD1c^+^	
		CLEC6A^+^	
		CLEC7A^+^	

## Tolerance Induction in the Thymus and Periphery

### Central Tolerance

Dendritic cells together with medullary thymic epithelial cells (mTECs) have a critical role in inducing central tolerance in the thymus by elimination of self-antigen-reactive thymocytes and generation of Tregs (2, 29). This is supported by the fact that mice lacking DCs show marked accumulation of CD4^+^ thymocytes without negative selection, leading to fatal autoimmunity (30). Three thymic DC subsets contribute to central tolerance: resident DCs (CD8α^+^ SIRPα^−^), migratory DCs (CD8α^−^ CD11b^+^ SIRPα^+^), and pDCs (CD11c^int^ CD45RA^int^). Resident DCs that develop from thymic lymphoid precursors are the most abundant subset (>50%) and are localized mainly in the medulla (31, 32). They contribute to the elimination of autoreactive thymocytes by presenting broadly expressed self-antigens and by cross-presenting both blood-derived antigens and tissue-specific antigens from mTECs (33, 34). On the other hand, migratory DCs and pDCs develop in the periphery and migrate to the corticomedullary perivascular space, which is freely permeable to circulating antigens *via* CCR2/α4 integrin and CCR9/α4 integrin, respectively (35, 36). Through this strategic location, migratory DCs and pDCs effectively capture and present blood-derived antigens. All of the DC subsets contribute to immune tolerance by presenting self-antigens and inducing negative selection of thymocytes with high affinity for self-antigens. Then, resident DCs provide immature T cells with a distinct self-antigenic repertoire, while migratory DCs and pDCs specialize in the presentation of peripheral antigens.

Thymic DCs are also important for the development of Tregs. Resident and migratory DCs are able to induce Tregs from thymocytes *in vitro* through different mechanisms (37, 38). Resident DCs promote Treg survival *via* their expression of CD70, while CD70-deficient migratory DCs effectively induce Tregs through an undefined pathway (38). Thymic stromal lymphopoietin (TSLP) expressed by Hassall’s corpuscles in the thymus medulla induces the tolerogenic phenotype on bone marrow-derived DCs, rendering them capable of converting naïve T cells into functional Tregs *in vitro* (39, 40). However, TSLP receptor-deficient mice have a normal number of Tregs in the thymus, suggesting that TSLP signaling is not essential for Treg development (29, 41). Thymic pDCs can also induce Tregs (42, 43) that are more efficient producers of IL-10 than those induced by other thymic DCs. These findings show that all of the DC subsets in the thymus are essential for the maintenance of central tolerance.

Recently, it has been examined how mTECs and CD8α^+^-resident DCs contribute to thymic tolerance using mice depleted of mTECs and/or resident DCs (44). Although mice depleted of resident DCs were normal and those depleted of mTECs developed liver inflammation, depletion of both resident DCs and mTECs resulted in multiorgan autoimmunity. Depletion of mTECs significantly reduced the production of thymic Tregs, but there was no additional effect on thymic Tregs when both mTECs and resident DCs were absent. Both CD4^+^ and CD8^+^ T cells in the thymus were increased in mice depleted of both mTECs and resident DCs. These results suggest that mTECs and resident DCs act to prevent autoimmunity through thymic T cell depletion in a cooperative manner, whereas mTECs have a non-redundant role in the production of thymic Tregs. Thus, mTECs and resident DCs have a unique role in tolerance induction that cannot be compensated for by remaining migratory DCs and pDCs.

The lymphotoxin β receptor (LTβR), a member of the TNF receptor superfamily, is a key regulator of thymic microenvironments and intrathymic tolerance, and its expression is detectable in multiple mTEC subsets (45, 46). The relationship between LTβR and coordination of mTECs and DCs for negative selection and Treg development has been recently investigated using LTβR-deficient mice (47). In LTβR-deficient mice, the thymic DC pool size was decreased due to reduced numbers of both pDCs and thymic cDCs, especially migratory DCs. In addition, LTβR-deficient mice showed a greater reduction in the numbers of CD4^+^CD8^−^ thymocytes and caspase-3^+^CD5^+^CD69^+^ thymocytes, representing cells undergoing negative selection, although they showed no change in Treg generation relative to control mice. These findings indicate that LTβR controls thymic tolerance by regulating the frequency and makeup of intrathymic DCs required for effective thymocyte negative selection rather than Treg generation.

### Peripheral Tolerance

**Figure 2:**
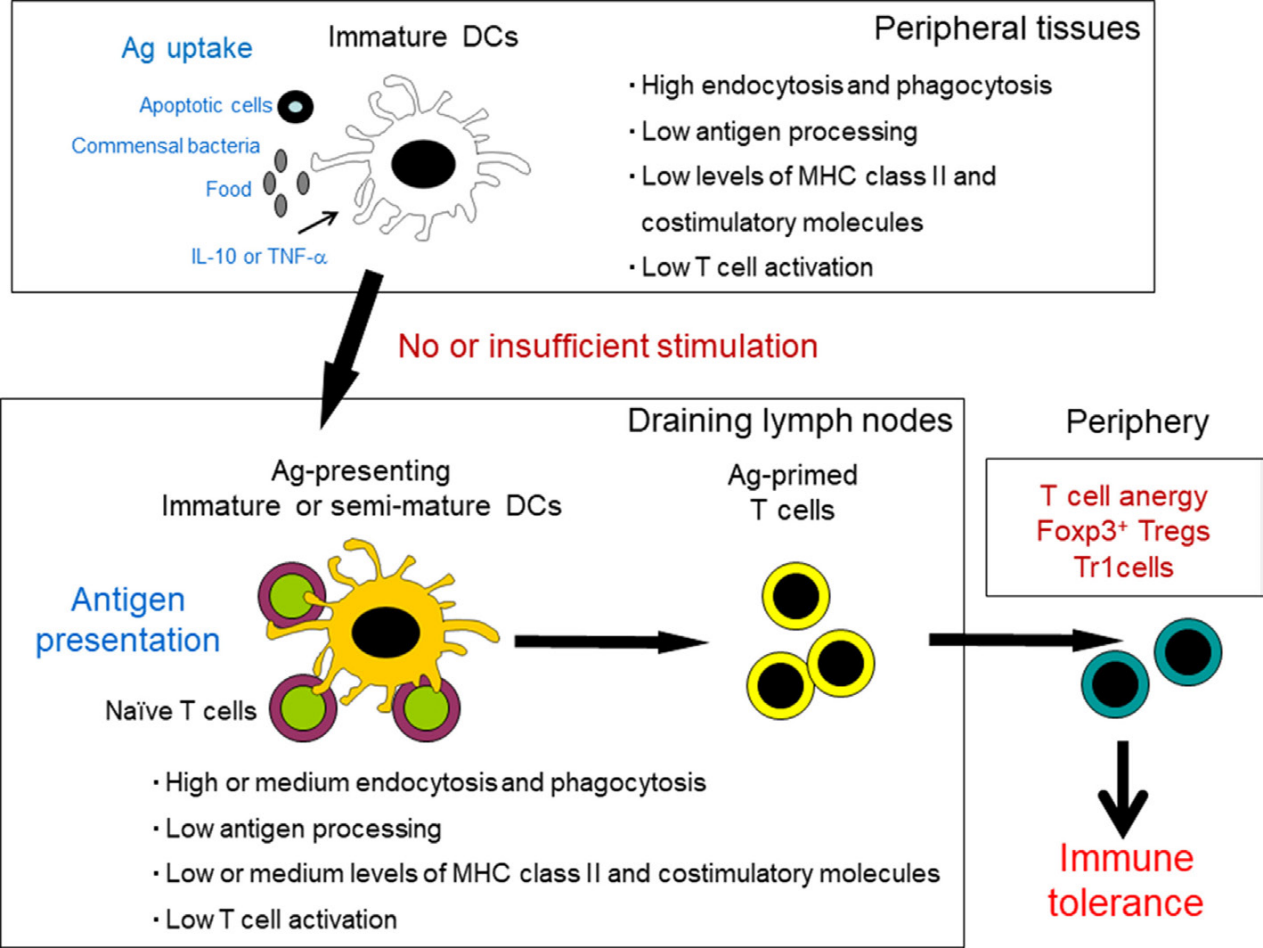
Induction of tolerance by peripheral dendritic cells (DCs) under steady-state conditions. Tissue-resident, immature DCs capture self-antigens from apoptotic cells, commensal bacteria, and food antigens. Under steady-state conditions, these DCs migrate to the draining lymph nodes without sufficient maturation. Antigen-presenting immature or semi-mature DCs provide insufficient stimulatory signals for T cells and therefore drive naïve T cells to anergy and differentiation into regulatory T cells and regulatory type 1 T (Tr1) cells.

Although thymic selection efficiently removes most self-antigen-reactive T cells, some remain and migrate into the periphery. Therefore, peripheral tolerance is crucial for maintenance of immune homeostasis throughout life. Tregs of thymic origin and peripheral DCs are crucial in inducing tolerance to antigens under steady-state conditions (Figure 2) (1, 48). The tolDC population consists of iDCs (naïve DCs) and alternatively activated DCs (semi-mature) that exhibit resistance to maturation in the presence of an inducing signal (5, 48). iDCs derived from bone marrow constitutively migrate throughout the periphery and lymphatic systems and become distributed in peripheral tissues. iDCs are poorly immunogenic as they show low surface expression of costimulatory molecules and have only modest levels of MHC class II (1, 48). A major functional characteristic of iDCs is their capacity for endocytosis and phagocytosis, including both foreign antigens and apoptotic cells, which occurs continuously in the steady state. The maintenance of DCs in an immature state, due to the absence of maturation stimuli, is associated with tolerance through induction of T cell deletion, anergy, and polarization toward a regulatory phenotype (4). Antigen-loaded iDCs in draining secondary lymphoid organs are more effective at inducing antigen-specific Treg populations than lymphoid-resident DCs *in vivo* (49). This supports a role for migratory iDCs in promoting peripheral tolerance under steady-state conditions. Furthermore, repetitive stimulation of T cells with iDCs can convert naïve T cells to Tregs (50, 51). Uptake of apoptotic cells polarizes DCs to a tolerogenic state, resulting in the promotion of T cell anergy and death and induction of Tregs *via* TGF-β1 secretion (52, 53). These data indicate that apoptotic cells are likely an insufficient stimulus for full DC maturation.

Dendritic cell subsets that differentiate through TLR ligands or in a specific cytokine environment might have involvement in tolerance, rather than in T cell activation (1, 48). This DC type has a semi-mature phenotype with reduced expression of MHC class II and costimulatory molecules in comparison to fully mDCs. Semi-mDCs differentiate in the presence of IL-6 or by stimulation with TLR ligands at low concentrations (54, 55). Stimulation of iDCs with TLR2 or TLR4 ligands at low concentration with the commensal bacterium *Bacteroides vulgatus*, which colonizes the intestinal tract, leads to secretion of IL-6, but not IL-12 or TNF-α (56). These DCs themselves differentiate into semi-mDCs through an autocrine loop, and exposure of iDCs to IL-6 (paracrine loop) triggers their differentiation to semi-mDCs. Furthermore, tolerogenic semi-mDCs are induced in the presence of IL-10 or TNF-α alone (57–60).

Several studies have demonstrated which DC subtypes contribute to peripheral Treg induction by combining methods of antigen delivery to DCs with diverse genetic mouse models lacking specific DC subtypes (1). Targeting of antigens to CD8α^+^/CD103^+^ DCs using recombinant chimeric antibodies such as DEC205, CLEC9A, and langerin results in the induction of peripheral Tregs (49, 61–63). Moreover, peripheral Treg induction is impaired through a reduction in the proportion of CD8α^+^/CD103^+^ DCs in BATF3-deficient mice and IRF8-deficient mice; both are transcription factors that are required for the development of CD8α^+^/CD103^+^ DCs (64, 65). In contrast, Treg induction is restored in mice deficient in IRF4, a transcription factor that governs CD11b^+^ DCs development. These data indicate that CD8α^+^/CD103^+^ DCs rather than CD11b^+^ DCs contribute to peripheral Treg induction.

Tolerogenic DCs show expression of immunomodulatory molecules and produce immunosuppressive factors such as IL-10, TGF-β, IL-35, and indoleamine 2,3-dioxygenase (IDO), resulting in T cell anergy and apoptosis and induction of Tregs (2, 48). The following section outlines these mechanisms.

## Mechanisms of Immune Tolerance by DCs

### T Cell Anergy

Anergy is a hyporesponsive state in which T cells remain inactive under conditions where immune activation would be undesirable, thus ensuring recognition of self-antigens and maintenance of a steady state (66). Anergy is induced in T cells that recognize antigen in the absence of costimulatory signals resulting from binding of CD28 on their surface to its ligand, CD80/CD86, on DCs. Consequently, IL-2 production is blocked, and T cells are unable to proliferate the same antigen (5, 67). Anergy can also be induced by coinhibitory signals such as programmed cell death-1 (PD-1) receptor and cytotoxic T lymphocyte antigen 4 (CTLA-4) (68, 69). PD-1 binds to PD-1 ligand (PD-L1) and PD-L2 on DCs, whereas CTLA-4 interacts with CD80/CD86 on DCs.

Several studies have shown that tolDCs can induce antigen-specific anergy through various mechanisms (70–73). tolDCs generated with IL-10 induced hyporesponsiveness of tetanus toxin (TT)-specific CD4^+^ T cell clone toward restimulation with TT-pulsed DCs (70). This inhibition of T cell proliferation was due not to release of soluble inhibitor factors from tolDCs but to a cell contact mechanism. Tuettenberg et al. have demonstrated that induction of anergy in CD4^+^ T cells by IL-10-modulated tolDCs was based on cell-to-cell contact through interaction of inducible T-cell costimulatory (ICOS)–ICOS ligand (ICOS-L) (71). Torres-Aguilar et al. showed that tolDCs generated with different combinations of the cytokines IL-10, TGF-β, and IL-6 induced anergy of TT-specific CD4^+^ T cells through thrombospondin-1 expression and production of prostaglandins and adenosine by tolDCs (72). Recently, Rodriguez et al. have reported that interaction of the dendritic cell-specific intercellular adhesion molecule-3-grabbing non-integrin with pathogens triggers specific signaling events that modulate DC maturation and activity, resulting in induction of T-cell anergy (73).

Induction and maintenance of T-cell anergy depend on activation of ubiquitin ligases of E3 family: Casitas B-lineage lymphoma-b (Cbl-b), Itchy homolog E3 ubiquitin protein ligase (Itch), and gene related to anergy in lymphocytes (GRAIL) (74). These enzymes act mainly through induction of proteolysis of molecules involved in TCR signaling (66, 67). T cells from Cbl-b- or Itch-deficient mice were hyperreactive and produced an increased amount of IL-2 (75–78). GRAIL was upregulated in anergic CD4^+^ T cells (79). In addition, the expression of these ubiquitin ligases in anergic T cells is associated with transcriptional factors, early growth response (Egr) type 2 and 3 (80). Blockade of Egr2 and Egr3 is resistant to anergy induction, while the transgenic expression of these factors suppresses TCR signaling (81, 82). In addition, anergic T cells could also act as Tregs and IL-10-producing Tr1 cells (83–86).

### Clonal Deletion

Clonal deletion, which involves the elimination of T cells through apoptosis, is an important process for maintenance of self-tolerance in the periphery (87). Apoptotic pathways can be triggered by extrinsic (receptor-dependent) and intrinsic (mitochondria-dependent) stimuli (88, 89). Both pathways involve a cascade of caspases whose activation commits cells to a death outcome. The extrinsic apoptosis pathway is initiated by binding of death receptors such as Fas and TNF receptor. Ligation of FasL, TNF, or TNF-related apoptosis-inducing ligand (TRAIL) to death receptors results in activation of caspase 8 and downstream caspases and, ultimately, cell death. tolDCs can also induce naïve and memory T cell apoptosis through interaction between FasL and Fas (90), TRAIL interaction with TRAIL receptors (91), and tryptophan catabolism due to IDO production (92, 93).

Tolerogenic DCs induce extensive T cell apoptosis in a manner dependent on interaction between DC FasL and Fas expressed by the target lymphocytes. Recently, a new immuno-suppressive action of DCs through the Fas signal has been reported (94). Splenic stroma-educated tolDCs expressed a high level of Fas, and Fas ligation was able to promote the inhibition of CD4^+^ T cell proliferation by tolDCs more significantly. Furthermore, Fas ligation preferentially induced tolDCs to produce IL-10. In addition, activated T cells promoted the secretion of more IL-10 by tolDCs through FasL. This shows that, at least from activated T cells, the Fas signal can promote the immunosuppressive action of Fas-expressing tolDCs, providing a new path for regulation of adaptive immunity by tolDCs. The cellular and molecular mechanisms of Fas-independent apoptosis of T cells induced by DCs have also been investigated by *in vitro* and *in vivo* analyses in MRL/*lpr* mice (95). This has revealed that FAS-independent T cell apoptosis can be induced by direct interaction between TRAIL receptor 2 on T cells and TRAIL on Fas-deficient DCs in MRL/*lpr* mice.

Indoleamine 2,3-dioxygenase is a rate-limiting enzyme that catalyzes the degradation of tryptophan into various metabolites, which subsequently inhibit T cell proliferation by impairing the cell cycle machinery and promoting apoptosis (48, 92, 93, 96, 97). IDO is not expressed constitutively in DCs and requires induction by various mediators including IFN-γ, TGF-β, and endotoxin (97). In rodents, CD103^+^ DCs in mesenteric lymph nodes (MLNs) and intestinal mucosa are known to express IDO. When IDO activity is inhibited, Th1 and Th17 cells are induced *in vivo*, preventing the development of Tregs that are specific for oral antigens (98). In contrast, tryptophan starvation increases the expression of the inhibitory receptors, immunoglobulin-like transcript 3 (ILT3) and ILT4, on DCs, leading to upregulation of Treg function. This phenomenon is associated with the GCN2 kinase-mediated stress response pathway (99).

Galectins, a family of β-galactoside-binding proteins, are expressed on DCs and also induce apoptosis of T cells (100–103). Especially, galectin 9 preferentially induces apoptosis of activated CD4^+^ T cells through the calcium–calpain–caspase 1 pathway (101). Galectin 9 is a ligand of T cell immunoglobulin- and mucin domain-containing molecule 3 (Tim-3) expressed in Th1 cells, and the galectin 9-induced cell death in Th1 cells is dependent on Tim-3 (104).

The intrinsic apoptosis pathway can be triggered by various stimuli such as gamma irradiation, pathogens, steroid hormone, and reactive oxygen radicals and by costimulatory blockade with CTLA-4 (88, 89, 105, 106). This pathway is induced by a change in mitochondrial membrane potential provoked by the Bcl-2 family of proteins (89). Cytochrome *c* is then released by the mitochondria, binds to the apoptotic protease-activating factor 1, and forms an apoptosome that triggers the activation of caspase 9, leading to cell death. Bcl-x_L_ and Bcl-2 impair intrinsic apoptosis by maintaining mitochondrial integrity (88). It has been reported that Bcl-x_L_ transgenic mice were resistant to induction of transplantation tolerance through costimulatory blockade, whereas a Bcl-2/Bcl-x_L_ inhibitor (ABT-737), in combination with costimulatory blockade and donor bone marrow cells, induced complete peripheral deletion of alloreactive T cells (105, 107, 108). On the other hand, the Bcl-2 family protein Bim present on mitochondrial membranes is involved in TCR-induced apoptosis, since deficiency of Bim impairs apoptosis of autoreactive thymocytes and mature T cells (109, 110). Taken together, these findings indicate that the intrinsic apoptosis pathway plays a critical role in not only peripheral T-cell homeostasis but also central tolerance.

### Induction of Tregs

Tolerogenic DCs can induce several subtypes of Tregs such as CD4^+^CD25^+^Foxp3^+^ T cells and Tr1 cells. This can be achieved through a number of mechanisms, including direct cell–cell contact-dependent signaling *via* surface molecules, as well as by alteration of Treg fate *via* secretory proteins (3). DCs are known to mediate Treg generation *via* several surface molecules, including CD80/CD86 (111, 112), ICOS-L (113), ILT3, and ILT4 (114) and PD-L1 or PD-L2 (115–117). Tolerance can be induced by presentation of MHC class II antigen by DCs without any additional costimulatory signal such as CD80/CD86 and ICOS-L or in combination with a coinhibitory signal such as PD-L1/2 and ILT3/4. Furthermore, ligation of CD80/CD86 by CTLA-4 drives Treg differentiation, whereas insufficient ligation of CD80/CD86 by CD28 leads to tolerance induction. ICOS-L expressed by DCs binds to its receptor on T cells and maintains the homeostasis of Tregs.

Recently, it has been demonstrated that DCs require B- and T-lymphocyte attenuator (BTLA), an immunoglobulin domain superfamily protein, to induce Tregs (64). BTLA is specifically expressed in DEC205^+^CD8α^+^ DCs. Anti-BTLA antibody, which prevents BTLA binding to its ligand, the herpes virus entry mediator (HVEM), expressed on T cells, dramatically reduces Treg conversion. In addition, in BTLA-deficient mice, Treg induction is also decreased. BTLA mediates the upregulation of CD5 expression in T cells through HVEM engagement-increased phosphorylation of mitogen-activated protein kinase kinase (MEK). MEK increases the expression of the Cd5-positive regulator ETS1 and inhibits the expression of the Cd5-negative regulator TCF-3. CD5 is expressed on all T cells and is a well-established negative regulator of TCR signaling. CD5 promotes Treg conversion in response to self and tolerizing peripheral antigens by blocking the activation of mechanistic target of rapamycin (118).

Dendritic cells secrete many factors that are known to induce tolerance and Treg generation. IL-10, produced in the surrounding milieu under tolerogenic conditions, can trigger the development of iDCs into semi-mature tolDCs in peripheral tissues. In turn, these tolDCs acquire the ability to generate IL-10 and migrate to neighboring lymphoid organs, where IL-10 produced by DCs regulates the development and proliferation of CD4^+^CD25^+^Foxp3^+^ T cells and Tr1 cells (48, 117). IL-10 also plays a pivotal role in regulating the expression of immune-inhibitory molecules. IL-10 upregulates the surface expression of ILT3 and ILT4 (114), PD-L1 (119), and CD95L (120) on DCs, leading to regulatory function and apoptosis.

TGF-β promotes the conversion of peripheral naïve T cells to Tregs through induction of Foxp3 expression (121). Similarly, several studies have demonstrated that DCs promote extrathymic Treg differentiation in a TGF-β-dependent manner (72, 122). Inhibition of T cell-specific TGF-β signaling *via* expression of a dominant-negative TGFβRII blocks the differentiation of Tregs (62). Coculture of Tregs with DCs results in secretion of IL-10, IL-27, and TGF-β by DCs, leading to the differentiation of Tr1 cells (123). DC-derived IL-27 suppresses the secretion of IL-1β and IL-23, induces the production of IL-10, and blocks Th17 differentiation (124). Through activation of STAT1 and STAT3, DC-derived IL-27 drives the transcription of IL-10 and activates the IL-10 promoter, thus inducing Tr1 differentiation (125). Moreover, IL-27 induces expression of the immunoregulatory molecule CD39, leading to suppression of T cell responses and autoimmunity (126). Gut-located DCs are a major source of RA, which promotes the generation of Tregs, while simultaneously inhibiting Th17 cells (127, 128).

Tolerogenic DCs secreted an anti-inflammatory cytokine, IL-35, and its production was enhanced upon stimulation with IFN-γ, LPS, or CD40 ligand (129). Conversely, IL-35 induced the conversion of cDCs to tolDCs (130). In addition to tolDCs, IL-35 is also secreted from Tregs and regulatory B cells (131–134). IL-35 is a member of the IL-12 family, consisting of IL-12α subunit p35 and IL-27β subunit Epstein–Barr virus-induced gene 3 and contributes to controlling homeostatic proliferation by suppressing T-cell proliferation and function (131, 132, 135). IL-35 could induce naïve T cells to differentiate into IL-35-producing Foxp3-induced Tregs, which maintain self-tolerance and promote infectious tolerance (131). IL-35 also plays an essential role in the balance between Th17 cells and Tregs through suppression of Th17 differentiation (132, 136). Moreover, a recent study has reported that mice vaccinated with IL-35-producing DCs showed promotion of tumor growth and amelioration of autoimmune encephalitis (130). Taken together, these findings suggest that IL-35 plays a significant role in the regulation of immune tolerance.

Plasmacytoid DCs induce Treg differentiation in the peripheral lymph nodes (137). Although, in the steady state, pDCs express very low levels of MHC class II and costimulatory molecules, activated pDCs upregulate MHC class II and migrate to the T cell area to induce Treg generation. Type I IFN and IL-10 produced by pDCs contribute to Treg generation. pDCs can also produce IDO and express PD-L1, and this is correlated with an increase of Treg numbers (115, 138).

Regulatory T cells are also able to affect DC function. Mutual interaction between DCs and Tregs is required for maintenance of immune tolerance: tolDCs induce Tregs, and conversely Tregs prepare DCs for an immunosuppressive role, thus extending the immunosuppressive function of Tregs. For example, IL-10 and TGF-β locally secreted from Tregs are able to suppress the maturation of DCs and render them tolerogenic (139). Another pivotal role of Tregs is their immunosuppressive effect when in contact with DCs. Recently, individual Treg–DC interaction events in lymph nodes have been examined *in vivo* using imaging techniques (140). Endogenous Tregs exhibited enhanced adhesion to antigen-presenting DCs, thus mediating the activation of conventional CD4^+^ T cells (T conv cells) in draining lymph nodes. Subsequent experiments using adoptive transfer of Tregs and MHC class II-deficient DCs have demonstrated that this increased Treg–DC adhesion can be promoted only by exposure to IL-2 without requiring MHC recognition. Importantly, physical contact with polyclonal Tregs significantly reduces the ability of DCs to form stable conjugates with cognate T conv cells *in vivo*, resulting in impaired T cell priming. These results suggest that Tregs of any TCR specificity can suppress DCs in a contact-dependent and MHC class II-independent manner. Moreover, the dynamic cytoskeletal components underlying contact-dependent Treg-mediated DC suppression have been analyzed using imaging (141). This revealed that Tregs, rather than T conv cells, exhibited strong intrinsic adhesiveness to DCs. This adhesion of Tregs caused sequestration of Fascin-1, an actin-bundling protein essential for the formation of immunological synapses and skewed Fascin-1-dependent actin polarization in DCs toward the Treg adhesion zone. This sequestration caused DCs to become lethargic, leading to reduced T cell priming. Mechanisms of immune tolerance by tolDCs are summarized in Table 2.

**Table 2:** Mechanisms of DC-induced immune tolerance.

T cell anergy
CD80/86-CTLA-4 interaction PD-L1/L2-PD-1 interaction ICOS-L–ICOS interaction Thrombospondin-1 expression Production of prostaglandins and adenosine Interaction of DC-SIGN with pathogens
**Clonal deletion (apoptosis)**
FasL–Fas interaction TRAIL–TRAIL receptor interaction Tryptophan catabolism *via* IDO production Galectin 9–Tim-3 interaction Intrinsic (mitochondria-dependent) apoptosis pathway
**Induction of Tregs**
PD-L1/L2–PD-1 interaction ICOS-L–ICOS interaction CD80/86–CTLA-4 interaction Expression of ILT3 and ILT4 Production of anti-inflammatory cytokines (IL-10, TGF-β, IL-27, and IL-35) BTLA–HVEM interaction Production of RA Tryptophan catabolism *via* IDO production IL-10 production by Fas-expressing tolerogenic DCs Plasmacytoid DCs function
**Other suppressive mechanisms**
Mutual interaction between DCs and Tregs

*CTLA-4, cytotoxic T lymphocyte antigen 4; PD-1, programmed cell death-1; PD-L1, programmed cell death-1 ligand; ICOS-L, inducible T-cell costimulatory ligand; DC-SIGN, dendritic cell-specific intercellular adhesion molecule-3-grabbing non-integrin; TRAIL, TNF-related apoptosis-inducing ligand; IDO, indoleamine 2,3-dioxygenase; Tim-3, T cell immunoglobulin- and mucin domain-containing molecule 3; ILT3, immunoglobulin-like transcript 3; BTLA, B- and T-lymphocyte attenuator; HVEM, herpes virus entry mediator; RA, retinoic acid; Tregs, regulatory T cells; DCs, dendritic cells*.

## Induction of Immune Tolerance by DCs in the Skin and Intestine

### Skin

The skin is the largest barrier organ separating the internal milieu from the external environment. It is exposed to not only physical stress but also a huge number of environmental antigens, including chemicals, commensal bacteria, and pathogens. Therefore, the immune system of the skin must detect and discriminate between these diverse antigens and induce appropriate tolerogenic or protective responses (142). The skin consists of two anatomically distinct layers, the epidermis and dermis, which are separated by a basement membrane. Langerhans cells (LCs), expressing the C-type lectin langerin (CD207), represent the sole tissue-resident DC population in the epidermis, while several subsets of DCs are resident in the dermis, including CD103^+^ cDCs, CD11b^+^ cDCs, and CD103^−^ CD11b^−^ cDCs. In addition, during inflammation, moDCs are recruited to the dermis (8). LCs are very motile, although most abundant in the spinous layer of the epidermis. LCs constantly migrate from the skin to draining lymph nodes even under steady-state conditions. In general, LCs induce effector-type immunity to pathogens and foreign proteins (143, 144). On the other hand, recent evidence suggests that LCs might be involved in peripheral tolerance induction. In a murine model of contact hypersensitivity (CHS), it has been demonstrated that the absence of LCs leads to an increase in the number of hapten-specific CD4^+^ and CD8^+^ T cells. This has revealed a mechanism of immune regulation in the skin whereby interplay with CD4^+^ T cells enables LCs to suppress antigen-specific responses through IL-10 production (145). Another CHS study involving experimental depletion and adoptive transfer has demonstrated that LCs confer protection against CHS development through a mechanism involving both anergy and deletion of allergen-specific CD8^+^ T cells and activation of a T cell population identified as ICOS^+^CD4^+^Foxp3^+^ Tregs (146). In a *Leishmania* infection model in mice, it has been demonstrated that the absence of LCs leads to reduced Treg immigration, indicating a suppressive role of epidermal LCs through promotion of Tregs (147). Recently, the use of a transgenic mouse model has facilitated analysis of the immune functions of LCs *in vivo* without any alteration in the complex composition of skin DC subsets (148). When ovalbumin was presented by steady-state LCs or by activated LCs, they developed antigen-specific CTL tolerance due to an increase in Tregs or the CTL memory response, respectively. This decision-making depends on the condition of the presenting LCs.

All dermal cDCs are derived from hematopoietic stem cell-derived progenitor cDCs, pre-cDCs, that continuously repopulate the dermis. Mainly, four subsets of cDCs are resident in the dermis: langerin^+^CD103^+^, langerin^−^CD11b^+^, langerin^−^CD11b^−^ and CD103^−^CD11b^−^ cDCs (8). Langerin^+^CD103^+^ cDCs include 10–20% dermal DCs and express XCR1. Langerin^+^CD103^+^ cDCs efficiently cross-present viral and self-antigens, and mice deficient in langerin^+^CD103^+^ show impaired priming of CD8^+^ T cells (18, 148, 149). On the other hand, langerin^+^CD103^+^ cDCs are capable of generating Tregs (150). When langerin^+^CD103^+^ cDCs were depleted in Lang-DTR mice, anti-DE205-mediated antigen-specific delivery to DCs was no longer able to induce antigen-specific Tregs, resulting in loss of immune tolerance (49, 150). Human BDCA-3^+^ DCs, the counterpart of murine langerin^+^CD103^+^ cDCs, have been shown to produce large amounts of IL-10 and to present self-antigens and induce Tregs (151). Langerin^−^CD11b^+^ cDCs include 70–80% dermal DCs. Langerin^−^CD11b^+^ cDCs can prime naïve CD4^+^ T cells to undergo Th2 differentiation and play a role in the immune response through IL-23/IL-17 signaling (152–156). RA-producing CD11b^+^ cDCs can induce Tregs upon migration to draining lymph nodes (157). Interestingly, it has recently been demonstrated that targeted deletion of IKKβ, a major activator of NF-κB, in DCs prevents the accumulation of skin migratory DCs in draining lymph nodes under steady-state conditions, thus compromising Treg conversion (158). Thus, NF-κB signaling appears to play a critical role in immunity and tolerance, as NF-κB is a key regulator of TLR-induced DC maturation and production of pro-inflammatory cytokines. Taken together, the evidence suggests that LCs and dermal DCs play a pivotal role in not only the immune response but also immune tolerance in the skin.

### Intestine

In the intestinal tract, IgA antibody production and the immune response by effector T cells are induced against pathogenic microorganisms. On the other hand, immune tolerance is also induced to avoid unnecessary inflammatory responses to food antigens and commensal bacteria. DCs play a critical role in the intestinal immune response and immune tolerance. In the intestinal mucosa, DCs are scattered diffusely throughout the intestinal lamina propria, within gut-associated lymphoid tissues including Peyer’s patches and solitary intestinal lymphoid tissues (SILT), and also in intestinal draining lymph nodes such as MLNs (159, 160). Migration of intestinal DCs plays an important role in immune surveillance and homeostasis of the gut. Migratory intestinal DCs can be derived from three different sites: the lamina propria, Peyer’s patches, and SILT presented within the small intestinal mucosa.

The murine small intestinal lamina propria contains at least three distinct populations of cDCs: CD103^+^CD11b^−^, CD103^+^CD11b^+^, and CD103^−^CD11b^+^ DCs. These three cDC subtypes are able to migrate *via* afferent lymphatics to the draining MLNs, a process that requires CCR7 signaling (161). cDCs in the lamina propria acquire antigens by handover, either from epithelial goblet cells or CX3CR1^high^ macrophages (162, 163). CD103^+^CD11b^+^ cDCs in the lamina propria migrate into the epithelium and capture pathogenic bacteria presented in the gut lumen by extending their dendrites (164). Upon antigen uptake, lamina propria cDCs enter the T cell zone of gut-draining MLNs for DC–T cell interaction. Double negative CD103^−^CD11b^−^ cDCs, which might exclusively originate from Peyer’s patches and SILT, have also been reported to carry antigens *via* afferent lymphatics to MLNs (165).

For adaptive immunity, migratory DC subsets derived from the lamina propria show some specialization in the generation of distinct Th cell subsets. CD103^+^CD11b^+^ cDCs activated with TLR produce high levels of IL-6 and induce IL-6-dependent Th17 differentiation, while CD103^+^CD11b^−^ and CD103^−^CD11b^+^ cDCs can drive Th1 differentiation rather than CD103^+^CD11b^+^ cDCs (165–168). On the other hand, cDCs in the lamina propria and MLNs, especially the population of CD103^+^ cDCs, play a central role in enforcing tolerance to food antigens and commensal bacteria under steady-state conditions. Many studies have shown that intestinal CD103^+^ cDCs highly induce Tregs through a mechanism mediated by TGF-β and RA (20, 159, 169). Tregs are induced by TGF-β, and RA enhances Treg induction only in the presence of TGF-β. TGF-β is secreted by intestinal CD103^+^ cDCs, Tregs, and intestinal stromal cells. Intestinal CD103^+^ cDCs highly express RALDH2, which convert vitamin A to RA, resulting in RA production from CD103^+^ cDCs. In addition to Treg induction, RA also induces gut-homing receptors, CCR9 for the small intestinal chemokine CCL25 and α4β7 integrin for the mucosal vascular addressin, MAdCAM1. In addition, it has recently been demonstrated that RA acts cell intrinsically in developing gut-tropic pre-mucosal DCs to trigger differentiation and drive the specialist role of intestinal CD103^+^CD11b^−^ and CD103^+^CD11b^+^ cDCs (170). Overall, the evidence suggests that through production of RA and TGF-β, intestinal CD103^+^ cDCs induce the differentiation of Tregs and home them into the intestinal mucosa to control tolerance.

A conditional knockout approach allowing the deletion of specific subsets of CD103^+^ cDCs has demonstrated that intestinal CD103^+^CD11b^−^ cDCs possess the greatest capacity to induce Treg differentiation, while CD11b^+^ DC subsets are rather inefficient (65). Moreover, PD-L1 and PD-L2 expression by MNL DCs has been implicated in the induction of oral tolerance *via* regulation of the Treg compartment (171). Comparison among four distinct DC subsets in MLNs—CD103^+^CD11b^+^PD-L1^high^, CD103^+^CD11b^−^PD-L1^high^, CD103^+^CD11b^−^PD-L1^low^, and CD103^−^CD11b^+^PD-L1^int^—has shown that CD103^+^CD11b^−^PD-L1^high^ DCs have a high capacity to induce Treg differentiation through TGF-β signaling (172). It has been reported that αvβ8 integrin, an activator of latent TGF-β, is expressed preferentially on CD103^+^CD11b^−^ cDCs in the lamina propria and MLNs and that αvβ8 integrin-expressing DCs induce Tregs *via* TGF-β activation (173). However, it remains unclear whether CD103^+^CD11b^+^PD-L1^high^ DCs and αvβ8 integrin-expressing DCs represent the same population. Under steady-state conditions, CD103^+^ cDCs in the lamina propria are tolerogenic. In contrast, under inflammatory conditions, CD103^+^ cDCs in the MLNs are immunogenic. MLN CD103^+^ cDCs from colitic mice have been shown to trigger Th1 responses with high levels of IL-6 production (174). During intestinal inflammation, MLN CD103^+^ cDCs acquire these pro-inflammatory properties with no ontogenetic changes. Therefore, as well as DCs in the skin, CD103^+^ cDCs in the intestine can also be immunogenic or tolerogenic due to the microenvironment.

**Figure 3:**
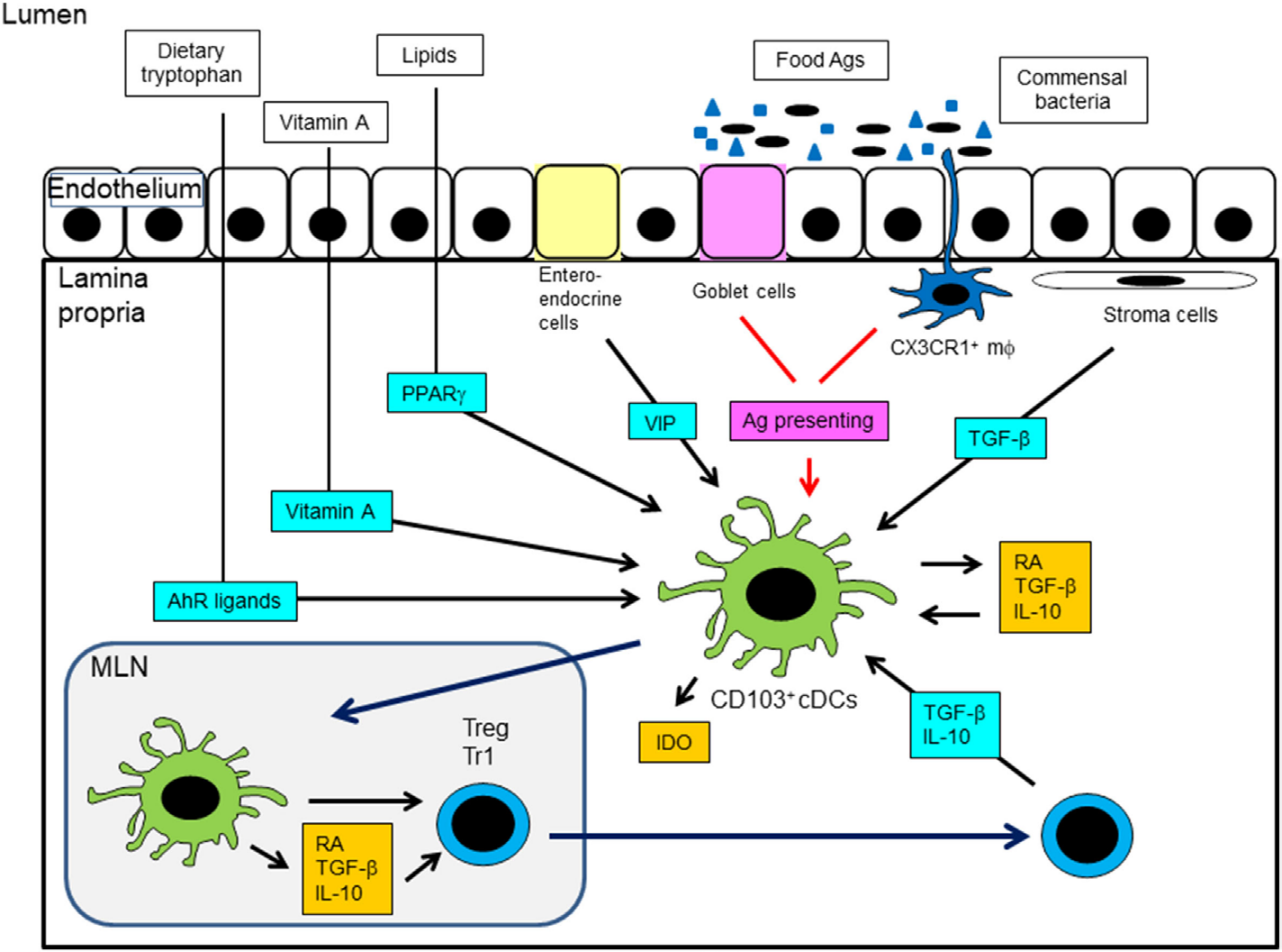
Mechanisms of induction of tolerogenic DCs in the intestine. MLN, mesenteric lymph node; AhR, aryl hydrocarbon receptor; PPARγ, peroxisome proliferator-activated receptor γ; VIP, vasoactive intestinal peptide; IDO, indoleamine 2,3-dioxygenase; RA, retinoic acid.

Some nutrients other than vitamin A are known to exert notable effects on intestinal tolerance. Tryptophan is a dietary element required for the IDO-dependent tolerogenic effects of intestinal DCs (175). Dietary tryptophan is metabolized into agonists for the aryl hydrocarbon receptor (AhR) through a series of cooperative biochemical reactions catalyzed by enzymes provided by gut commensal bacteria and the host (176). Tryptophan-derived AhR ligands induce the production of IL-10 and IL-27 by DCs, favoring the generation of Tregs and Tr1 cells. Diet-derived lipid mediators can activate peroxisome proliferator-activated receptor γ (PPARγ), and DCs exposed to PPARγ can be tolerogenic (7, 177). The gut mucosa is permeated by a complex nervous system and therefore exposed to the local release of neurotransmitters. Vasoactive intestinal peptide (VIP) is produced by intestinal enteroendocrine and immune cells and acts as a vasodilator and regulator of epithelial permeability. VIP suppresses LPS-induced DC maturation and promotes the differentiation of Tregs and Tr1 cells (178, 179). Taken together, these findings suggest that metabolites provided by the diet and gut flora act in concert with endogenous signals to regulate the ability of DCs to control T cell responses and tissue homeostasis. Mechanisms of induction of tolDCs in the intestine are summarized in Figure 3.

## Conclusion

Dendritic cells play a pivotal role in immune tolerance and homeostasis in the body. In this review, we present an overview of our current understanding of the mechanisms of tolerance induction by DCs in the body: DC origin, differentiation, and subsets; tolerance induction in the thymus and periphery; mechanisms of immune tolerance by DCs; and induction of immune tolerance by DCs in the skin and intestine. However, since analysis of the abovementioned mechanisms in health and disease is still insufficient, further studies are needed. A thorough understanding of the mechanisms that control immune tolerance will guide the development of novel strategies for the treatment of autoimmunity.

## Author Contributions

HH designed the study and drafted manuscript. TM edited the manuscript.

## Conflict of Interest Statement

The authors declare that the research was conducted in the absence of any commercial or financial relationships that could be construed as a potential conflict of interest.
